# Development of Split Ring Resonator Shaped Six Element 2 × 3 Multiple Input Multiple Output Antenna for the C/X/Ku/K Band Applications

**DOI:** 10.3390/mi14040874

**Published:** 2023-04-19

**Authors:** Meshari Alsharari, Vishal Sorathiya, Ammar Armghan, Kavan Dave, Khaled Aliqab

**Affiliations:** 1 Department of Electrical Engineering. College of Engineering, Jouf University, Sakaka 72388, Saudi Arabia; 2 Faculty of Engineering and Technology, Parul Institute of Engineering and Technology, Parul University, Waghodia Road, Vadodara 391760, Gujarat, India; vishal.sorathiya23995@paruluniversity.ac.in; 3 Department of Information and communication technology, Marwadi University, Rajkot 360005, Gujarat, India

**Keywords:** MIMO, split-ring resonator, gain, directivity, gigahertz, TARC, ECC, DG, CCL

## Abstract

In this manuscript, we have numerically investigated and experimentally verified the six-element split ring resonator and circular patch-shaped multiple input, multiple output antenna operating in the 1–25 GHz band. MIMO antennas are analyzed in terms of several physical parameters, such as reflectance, gain, directivity, VSWR, and electric field distribution. The parameters of the MIMO antenna, for instance, the envelope correlation coefficient (ECC), channel capacity loss (CCL), the total active reflection coefficient (TARC), directivity gain (DG), and mean effective gain (MEG), are also investigated for identification of a suitable range of these parameters for multichannel transmission capacity. Ultrawideband operation at 10.83 GHz is possible for the theoretically designed and practically executed antenna with the return loss and gain values of −19 dB and −28 dBi, respectively. Overall, the antenna offers minimum return loss values of −32.74 dB for the operating band of 1.92 to 9.81 GHz with a bandwidth of 6.89 GHz. The antennas are also investigated in terms of a continuous ground patch and a scattered rectangular patch. The proposed results are highly applicable for the ultrawideband operating MIMO antenna application in satellite communication with C/X/Ku/K bands.

## 1. Introduction

MIMO (multiple-input, multiple-output) antennas have emerged as an effective solution to enhance the wireless communication system’s capacity and reliability [1]. They are increasingly being used in GHz frequency applications [2], which demand high data rates and reliable communication links [3]. MIMO antenna systems provide higher throughput by transmitting multiple signals simultaneously using multiple antennas [4]. The number of antennas used for transmitting and receiving in a wireless communication system determined the total number of antennas utilized in a multiple-input multiple-output (MIMO) system. [5,6,7]. The GHz frequency range includes frequencies from 1 GHz to 30 GHz [8,9,10,11]. These frequencies are widely used in various applications, such as Wi-Fi [12], cellular communication [13], satellite communication [14], and radar systems [15]. MIMO antennas can be designed and optimized for GHz frequency applications to meet specific system requirements. The design of a MIMO antenna for GHz frequency applications involves considering various parameters such as operating frequency, bandwidth, polarization, gain, radiation pattern, and size. One of the most critical aspects of designing a MIMO antenna for GHz frequency applications is the operating frequency. The operating frequency should be selected based on the specific application requirements. For example, the most common Wi-Fi frequency bands are 2.5 GHz and 5 GHz, while cellular communication systems operate in the 700 MHz to 2.7 GHz frequency range. The operating frequency of the MIMO antenna is determined by the size and shape of the antenna elements [16,17]. The operating frequency and bandwidth of a MIMO antenna are heavily influenced by parameters such as the shape and size of the antenna, and on the basis of these parameters, antennas have been categorized into two distinct groups: (1) microstrip antennas [18,19] and (2) slot antennas [20,21]. Microstrip antennas are widely used in GHz frequency applications due to their compact size, small height and width, and compatibility with a wide range of electrical devices. A microstrip antenna has a ground plane and a conducting patch on opposite sides of a dielectric substrate. In addition, the conducting patch is usually fed using a coaxial cable [22] or a microstrip line [23]. In the microstrip antenna, the dimension of the patch decides the frequency of operation [24]. A smaller patch size results in a higher operating frequency, while a larger patch size results in a lower operating frequency. Slot antennas are another type of MIMO antenna used in GHz frequency applications. Slot antennas consist of a metal plate with a slot or a narrow opening in the center. The slot is usually excited using a microstrip line or a coaxial cable. Slot antennas are easy to fabricate and offer a wide bandwidth [25]. The frequency of operation of a slot antenna is directly dependent on the dimensions of the slot. A smaller slot size results in a higher operating frequency, while a larger slot size results in a lower operating frequency. The performance of MIMO antennas for GHz frequency applications is evaluated based on various parameters such as radiation pattern, gain, efficiency, and isolation. MIMO antennas can be designed to operate at different frequencies, depending on the specific application. In GHz frequency applications, the design of MIMO antennas requires careful consideration of the antenna’s physical dimensions and radiation properties to ensure optimal performance. The operating frequency of MIMO antennas in GHz applications can range from a few GHz up to several tens of GHz. One important consideration in designing MIMO antennas for GHz applications is the physical size of the antennas. At higher frequencies, the wavelength of the electromagnetic waves becomes shorter, and the size of the antennas must be proportionally reduced to achieve the desired performance. This can be challenging, as smaller antennas typically have lower gain and efficiency than larger antennas. As a result, MIMO antennas for GHz applications must be designed to optimize the trade-off between size, gain, and efficiency [26]. Another important consideration in designing MIMO antennas for GHz applications is the radiation pattern [27]. In general, MIMO antennas should radiate in a way that maximizes signal strength while minimizing interference between the different antenna elements. This requires careful design of the antenna elements and their placement within the MIMO array [26,28]. One common approach is to use directional antennas with a narrow beamwidth to maximize signal strength in the desired direction while minimizing interference with other antennas [29]. MIMO antennas for GHz applications can take a variety of forms, including patch antennas [30], dipole antennas [31], slot antennas [20], and others. Patch antennas are particularly well-suited for GHz applications due to their small size and ease of integration with other electronic components. In addition, patch antennas can be designed to have a wide impedance bandwidth, which allows them to operate over a broad range of frequencies.

In this manuscript, we have numerically investigated and experimentally verified the design of a six-element split-ring resonator and circular patch-shaped multi-input, multi-output antenna in the 1–25 GHz frequency range. MIMO antennas are analyzed in terms of several physical parameters, such as reflectance, gain, directivity, VSWR, and electric field distribution. In order to determine whether or not this antenna is suitable for multichannel transmission, we have also provided additional MIMO antenna characteristics, including envelope correlation coefficient, total active reflection coefficient, channel capacity loss, mean effective gain and diversity gain. This manuscript is majorly divided into three subdivisions. The first segments include the design and specification of all five stages of the proposed antenna. The second segment consists of measurement and simulation results and discussions of the proposed antenna. Finally, the last segment demonstrates the influence of the various antenna characteristics in a situation where numerous ports are excited simultaneously.

## 2. Design of the Six-Element MIMO Antenna Structure

**Figure 1:**
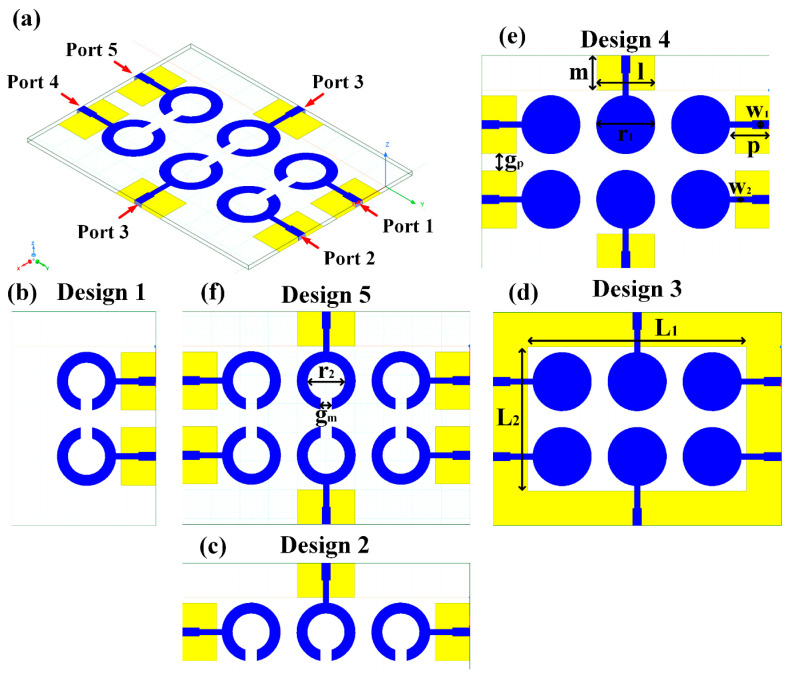
Schematic of the six-element, split-ring resonator-based MIMO antenna with different antenna designs. (**a**) Three-dimensional view of the proposed MIMO antenna with the notation of the input ports. The top and bottom layers of the antenna of copper are formed on the dual-layer FR4 substrate material. Schematic view of (**b**)Tow element split ring patch antenna with the rectangular patch (Design 1), (**c**) Three element split ring patch antenna (Design 2), (**d**) Circular patch shaped six element antennas with the continuous ground (Design 3), (**e**) Circular patch shaped six element antennas with a rectangular patch (Design 4), and (**f**) split ring resonator shaped six element antennas with rectangular patch.

The schematic of the six-element, split-ring resonator-shaped MIMO antenna is depicted in Figure 1. Figure 1a presents a 3D viewpoint of the proposed MIMO antenna, complete with input port labeling. On the dual-layer FR4 substrate material, copper is chosen as the material choice for the bottom as well as the top layer of the proposed structure. A schematic view of other two- and six-element antennas is shown in Figure 1b–e. Table 1 gives a complete idea of antenna dimensions. The overall antenna is in size of 74 × 100 mm^2^. We have performed numerical analysis on five distinct antenna designs that contain the radiation patch elements either on a split ring resonator or in a circular patch shape of the structure. In all the antenna designs, the ground is either continuous for all patches in the shape of a rectangle, or individual rectangular copper patches are placed over the individual MIMO elements, which can be seen in Table 2. The antennas are classified into five types as depicted in Table 2. Table 2 illustrates the simulated antenna design along with the fabricated antenna images and its structure description. The proposed structure is simulated using the commercially available software HFSS. The proposed antennas are designed and built with a variety of criteria in mind, including their reflectance coefficient, gain, directivity, and radiation pattern for the 1 to 25 GHz frequency range. A vector network analyzer (VNA) N9918A (30 kHz to 26 GHz) with a purpose-built antenna and electromagnetic compatibility (EMC) test anechoic chamber has been used to experimentally verify the suggested antennas. The mathematical equations and computational facility have been involved as per equations suggested in the upcoming results and discussion section of this manuscript. 

**Figure 2:**
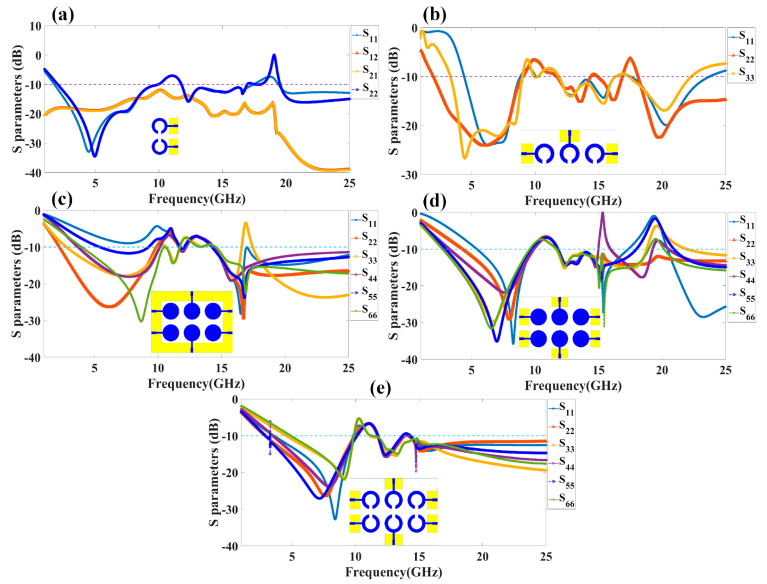
Numerically calculated reflectance coefficients for the different proposed MIMO antenna designs: (**a**) Design 1, (**b**) Design 2, (**c**) Design 3, (**d**) Design 4, and (**e**) Design 5. The results are presented in terms of the return loss parameters, such as S_11_, S_22_, S_33_, S_44_, S_55_, and S_66_. A dotted line is put for the reference to identify the reflectance <−10 dB.

**Figure 3:**
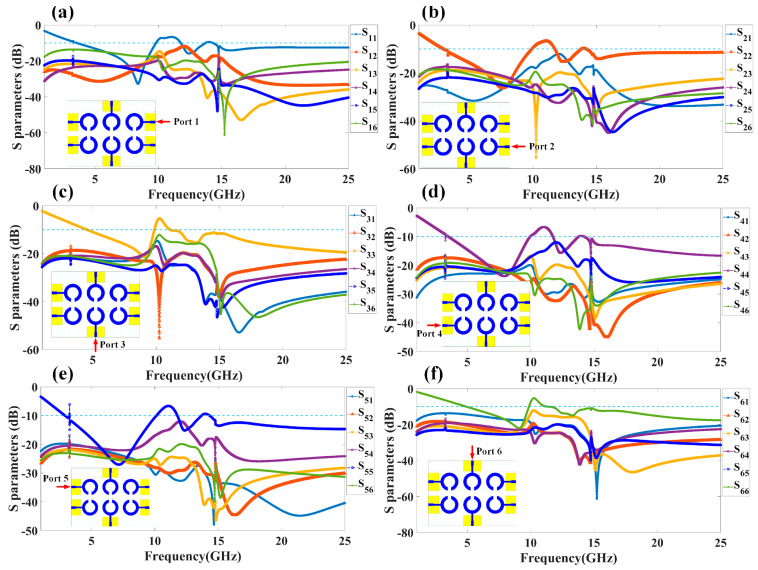
Numerically calculated reflectance coefficients for the different proposed MIMO antenna Design 5. The results are presented in terms of the S parameters, such as S_ij,_ where i, j = 1, 2, 3, 4, 5, and 6 for indication of specific reflection coefficient at port. The S parameters are calculated by considering excited at (**a**) Port 1, (**b**) Port 2, (**c**) Port 3, (**d**) Port 4, (**e**) Port 5, and (**f**) Port 6. Dotted line is put for the reference to identify the reflectance <−10 dB.

We have calculated the reflectance response of all five types of antennas by applying the excitation at all antenna ports. The reflectance response achieved through the numerical study is depicted in Figure 2. Figure 2a illustrates the reflectance response for the two-element split ring resonator-based antenna structure. The dotted line in Figure 2 for all the responses is considered for the identification of the values of the reflectance response below the level of <−10 dB. This range of the reflectance value helps us to identify the values of the resonating bands. We have calculated the overall resonating conditions where Sij < −10 dB, where i, j = 1, 2, 3, 4, 5, 6. The reflection conditions Sij < −10 dB allow us to choose the resonating band where co-port (port to port isolation) and cross-port reflectance (inter element isolation) are below the <10% for the effective antenna radiation of a specific frequency. Figure 2b shows how the S parameters change when the circuit is excited through three ports. Similarly, Figure 2c–e shows the variation in the co-port reflectance parameters for the six-port excitation in designs 3, 4, and 5 of the antenna structure, respectively. For Design 5 of the MIMO structure based on a split ring resonator, we have also included the computed S parameters for the multi-port excitation configurations. The results obtained for these port excitation conditions are shown in Figure 3. We can observe the values of the S parameters where the values of the reflection coefficients of the input signals for each of the six antenna elements, i.e., S_ij,_ where i and j refer to 1, 2, 3, 4, 5, and 6, are <−10 dB and other S parameters S_12_, S_13_, … are also <−10 dB. These conditions are satisfied for the identification of the overall resonating band where the co-port and cross-port reflectance values are <10% for the effective radiation. 

The bands derived from this calculation are shown in Table 3. We can identify that all the antennas are offering the minimum three operating bands, and the conditions of S_ij_ < −10 dB are satisfied. In Design 1 of the antenna, we observed the three operating bands with bandwidth values of 7.88 GHz, 5.17 GHz, and 5.46 GHz. This antenna covers an overall area of 74% of the 1–25 GHz band in the frequency spectrum for radiation conversion. The reflection coefficient’s minimum value is observed at −34.51 dB. The maximum peak gain observed is 12.64 GHz with a return loss of −16.11 dB and a band of 5.46 GHz. We can observe similar types of antenna operating bandwidth, peak gain, and bandwidth for all the antennas in Table 3. According to our measurements, the bandwidth of Design 5 is the highest in the frequency range of 14.32 to 25 GHz, which is 10.68 GHz. The minimum bandwidth of 1.38 is observed in Design 2 for 14.95 to 16.33 GHz of the frequency band. The maximum gain of 28.23 dBi is observed in the third band of antenna operation in Design 5 of the antenna. It is observed that 49% of the band is covered for radiation in Design 2 of the antenna structure. In designs 3, 4, and 5, we have observed that 70%, 46%, and 77% of the band are covered for radiation over 1 to 25 GHz of the spectrum. 

**Figure 4:**
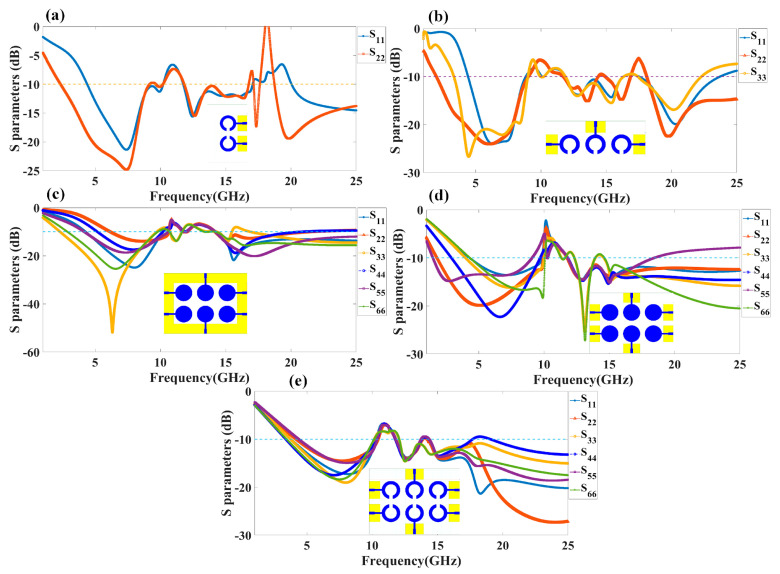
Measured values of reflectance coefficients for the different proposed MIMO antenna designs: (**a**) Design 1, (**b**) Design 2, (**c**) Design 3, (**d**) Design 4, and (**e**) Design 5. The results are presented in terms of the return loss parameters, such as S_11_, S_22_, S_33_, S_44_, S_55_, and S_66_. A dotted line is put for the reference to identify the reflectance <−10 dB.

We have fabricated the proposed structures, and the measurement results for the proposed structure are shown in Figure 4. Similar to the results, we have presented the S parameter results of the proposed structure for all the antenna designs. We have observed the variation in the measured results as compared to the simulations. The variation in both results is due to the connector and the loss. Gain values from simulation and measurement are also showing discrepancies. It could be because of dielectric losses, radiation inefficiency, reflection, and diffraction of waves in a real-world environment. The values of the S parameters and their associated frequency spectrum are shown in Table 4. We have observed that Design 1, after measurement results, offers three operating bands of 4.77 GHz, 3.84 GHz, and 6.1 GHz. The minimum return loss in Design 1 is observed to be −24.82 dB in the first band of operations. In Design 2, we have observed the maximum bandwidth of 5.9 GHz in the last band of 18.68 to 24.58 GHz with a return loss of −26.50 dB. Similarly, we have observed maximum bandwidths of 7.55 GHz, 4.77 GHz, and 5.95 GHz for designs 3, 4, and 5, respectively. We observed the maximum band of 5.95 GHz in the last design with return loss values of −27.40 dB. Overall, the structure of the six-element antenna with split ring resonator and rectangular ground patch offers the maximum bandwidth and minimum return loss for both simulated and measurement results. We have also observed in Design 3 and Design 4 that the number of resonating bands is increasing. In Design 3, the measurement results show 53% of the band of overall frequency converted to radiation mode. In Design 4, the number of bands increases, but overall, 47% of the bandwidth is converted to radiation mode. Out of all the structures measured, the maximum band of 68% converted to radiation conditions is for Design 5.

The split ring resonator shape provides advantages over a normalized patch structure in terms of compact size, metamaterial effect, broadband response, and flexibility of operation. The results shown in Figure 3 and Figure 4 show an improvement in bandwidth as compared to the normal patch structure design. The detailed bandwidth variations for different frequency slots can be observed in Table 3 and Table 4. It is also possible to tune these operating bands by changing the radius of the split ring resonator. Radiation losses may be minimized and overall efficiency increased by using split ring resonators instead of circular patches. Due to this, SRRs may be used in places such as radar systems and wireless power transmission where low-loss or high-efficiency performance is necessary.

**Figure 5:**
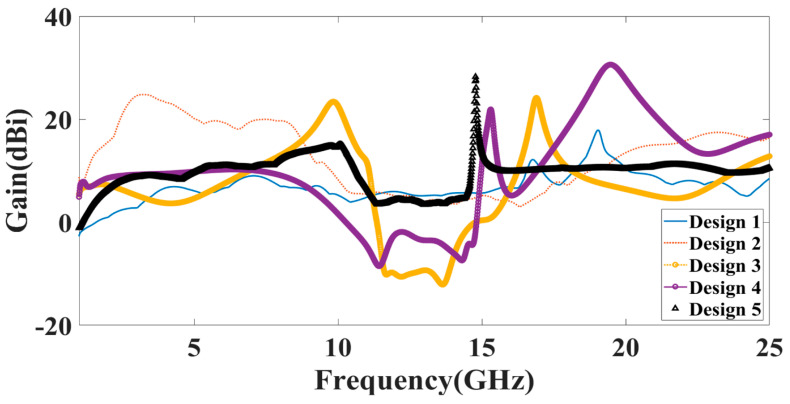
Calculated values of the gain for the different antenna design for the frequency range of 1 to 25 GHz.

**Figure 6:**
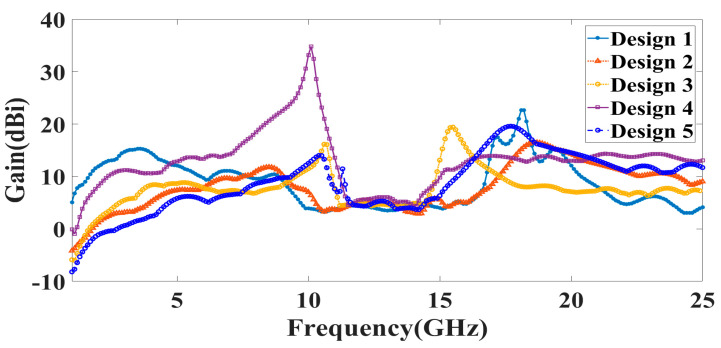
Measured values of the gain for the different antenna designs for the frequency range of 1 to 25 GHz.

**Figure 7:**
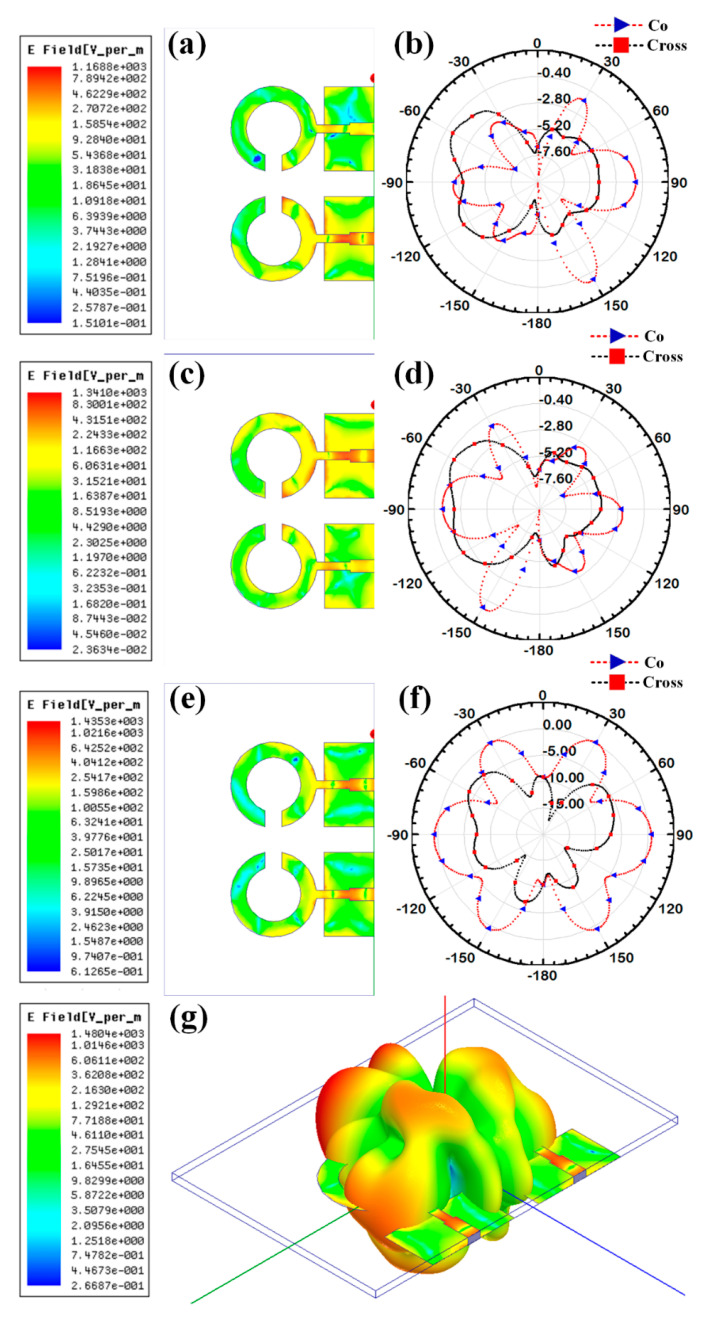
Electric field intensity and polar radiation pattern for the Design 1. Normalized electric field distribution for the different port excitation conditions such as (**a**) (P1 = 0, P2 = 1) (**c**) (P1 = 1, P2 = 0), and (**e**) (P1 = 1, P2 = 1). Co-polarization and cross-polarization 2D radiation pattern for the different port excitation conditions such as (**b**) (P1 = 0, P2 = 1), (**d**) (P1 = 1, P2 = 0), and (**f**) (P1 = 1, P2 = 1). (**g**) three-dimensional radiation pattern along with the electric field distribution for the (P1 = 1, P2 = 1) condition.

**Figure 8:**
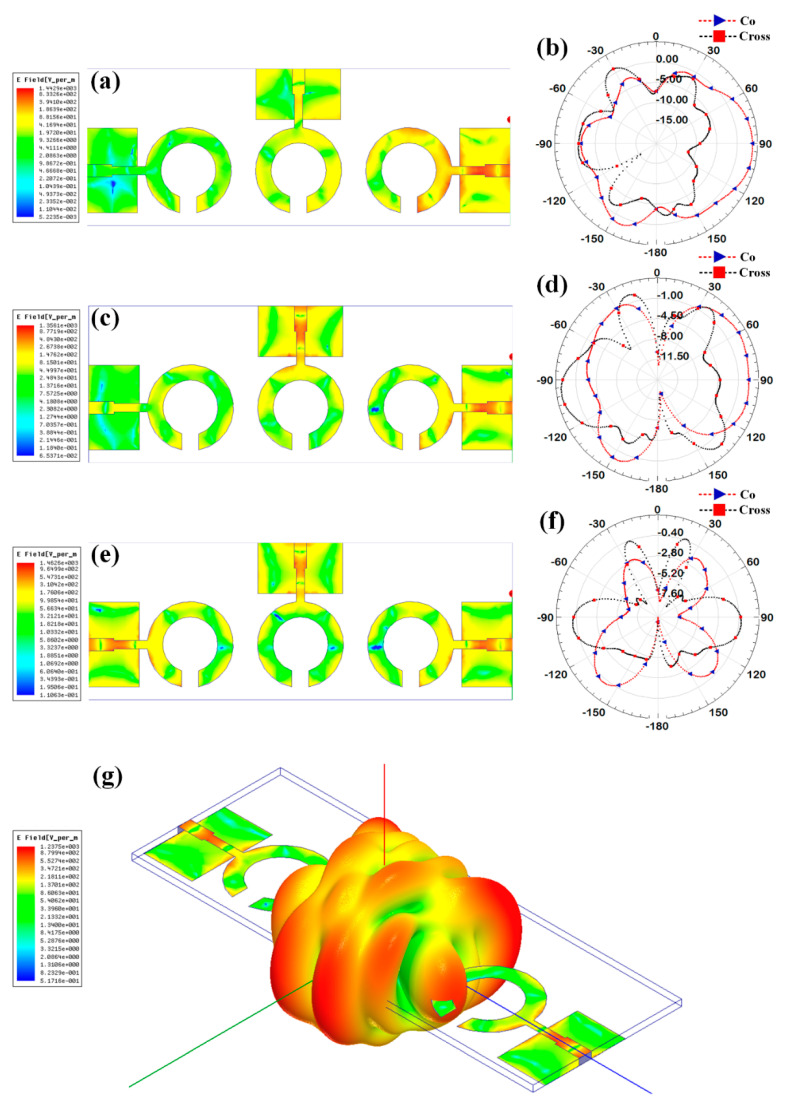
Electric field intensity and polar radiation pattern for the Design 2. Normalized electric field distribution for the different port excitation conditions such as (**a**) (P1 = 1, P2 = 0, P3 = 0), (**c**) (P1 = 1, P2 = 1, P3 = 0), and (**e**) (P1 = 1, P2 = 1, P3 = 1). Co-polarization and cross-polarization 2D radiation pattern for the different port excitation conditions such as (**b**) (P1 = 1, P2 = 0, P3 = 0), (**d**) (P1 = 1, P2 = 1, P3 = 0), and (**f**) (P1 = 1, P2 = 1, P3 = 1). (**g**) three-dimensional radiation pattern along with the electric field distribution for the (P1 = 1, P2 = 1, P3 = 1) conditions.

**Figure 9:**
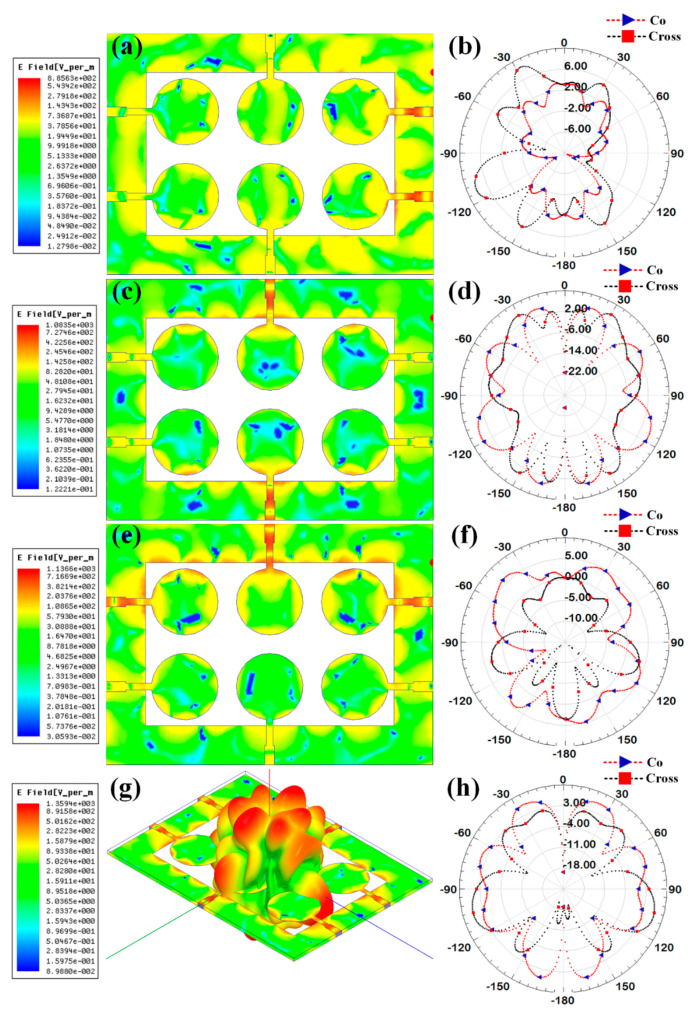
Electric field intensity and polar radiation pattern for the Design 3. Normalized electric field distribution for the different port excitation conditions such as (**a**) (P1 = 1, P2 = 1), (**c**) (P3 = 1, P6 = 1), and (**e**) (P1 = 1, P5 = 1, P6 = 1). Co-polarization and cross-polarization 2D radiation pattern for the different port excitation conditions such as (**b**) (P1 = 1, P2 = 1), (**d**) (P3 = 1, P6 = 1), and (**f**) (P1 = 1, P5 = 1, P6 = 1). (**g**) three-dimensional radiation pattern along with the electric field distribution for the all-port excitation conditions. (**h**) Co polarization and cross polarization 2D radiation pattern for all port excitation conditions.

**Figure 10:**
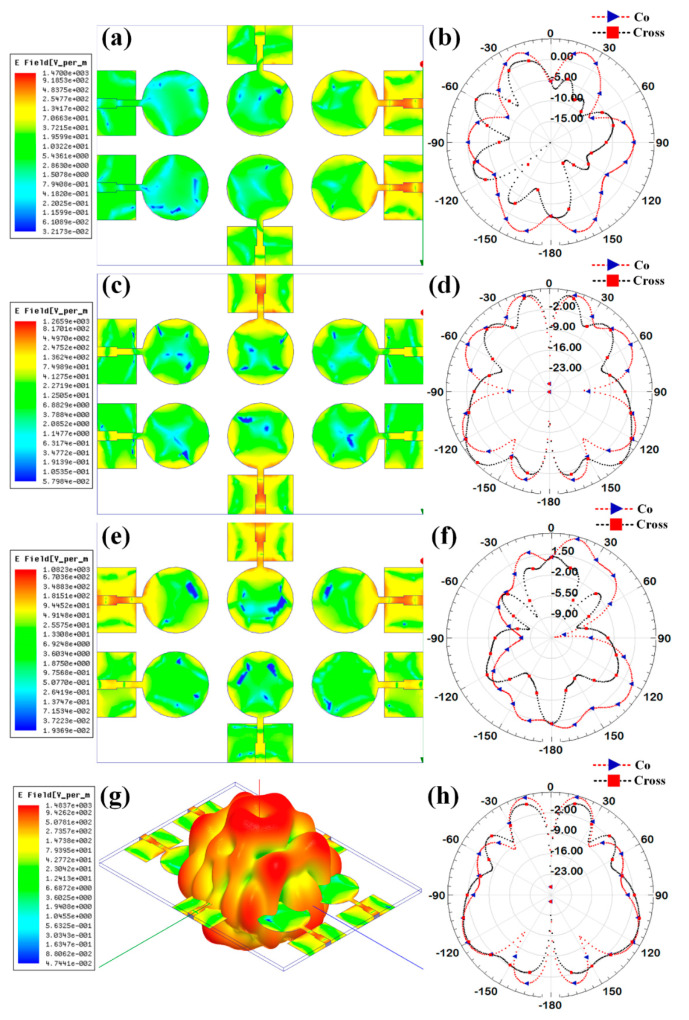
Electric field intensity and polar radiation pattern for the Design 4. Normalized electric field distribution for the different port excitation conditions such as (**a**) (P1 = 1, P2 = 1), (**c**) (P3 = 1, P6 = 1), and (**e**) (P1 = 1, P5 = 1, P6 = 1). Co-polarization and cross-polarization 2D radiation pattern for the different port excitation conditions such as (**b**) (P1 = 1, P2 = 1), (**d**) (P3 = 1, P6 = 1), and (**f**) (P1 = 1, P5 = 1, P6 = 1). (**g**) three-dimensional radiation pattern along with the electric field distribution for the all-port excitation conditions. (**h**) Co polarization and cross polarization 2D radiation pattern for all port excitation conditions.

**Figure 11:**
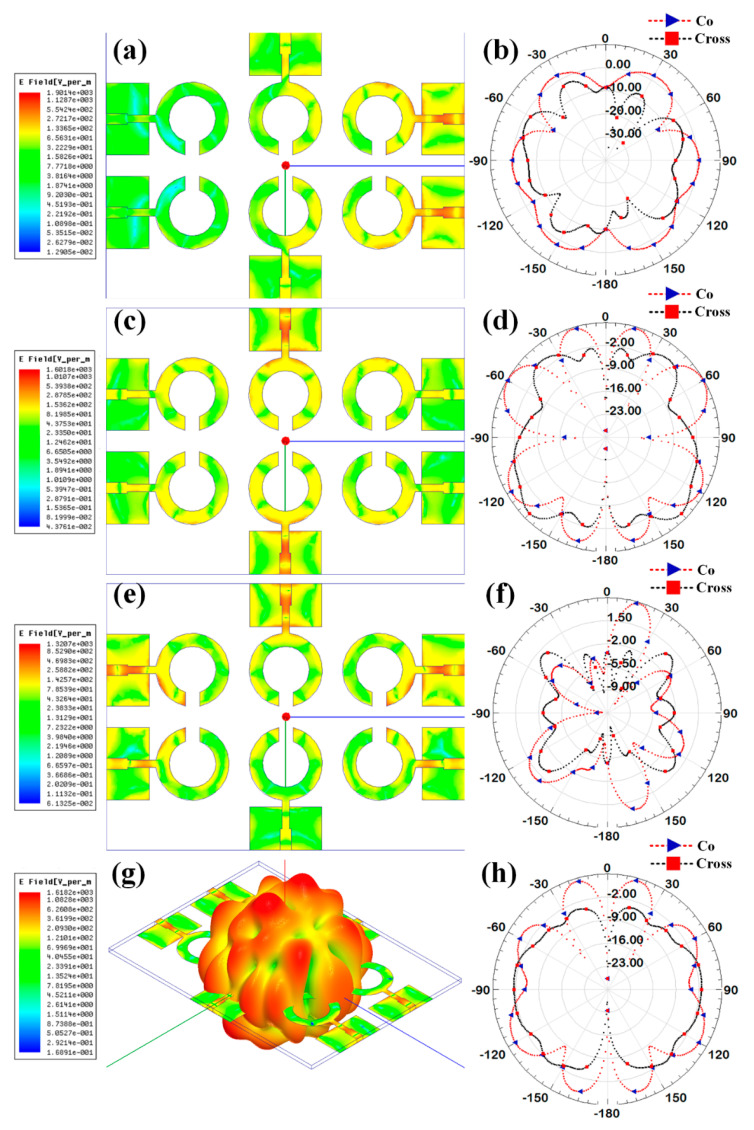
Electric field intensity and polar radiation pattern for the Design 5. Normalized electric field distribution for the different port excitation conditions such as (**a**) (P1 = 1, P2 = 1), (**c**) (P3 = 1, P6 = 1), and (**e**) (P1 = 1, P5 = 1, P6 = 1). Co-polarization and cross-polarization 2D radiation pattern for the different port excitation conditions such as (**b**) (P1 = 1, P2 = 1), (**d**) (P3 = 1, P6 = 1), and (**f**) (P1 = 1, P5 = 1, P6 = 1). (**g**) three-dimensional radiation pattern along with the electric field distribution for the all-port excitation conditions. (**h**) Co polarization and cross polarization 2D radiation pattern for all port excitation conditions.

Additionally, the gain over the frequency range of 1–25 GHz is presented as the second antenna parameter for identifying the antenna’s efficient operation. Gain values for each suggested antenna design have been computed and shown in Figure 5. Gain variations from −15 dBi to +30 dBi are visible. Out of all the results of the gain with the specified antenna radiation spectrum, we need to think about the effective gain values of each antenna structure. In the specific radiation bands shown in Table 3, the gain profile is considered for the observations. We have identified the peak gain values for the specific antenna and specific radiation bands. These results of gain are shown in Table 3. The derived values of gain from a fabricated prototype are presented in Figure 6 for the entire frequency spectrum. The associated peak gain and bandwidth values are represented in Table 4 for a suitable comparison of the derived resonating frequency bands for different antenna designs. The radiation pattern of the antenna and intensity of the normalized electric field for the multi-port excitation conditions are derived, and the results are presented in Figure 7, Figure 8, Figure 9, Figure 10 and Figure 11. Figure 7 shows the changes in electric field for the multi-port excitation conditions such as (P1, P2 = (0, 1)), (P1, P2 = (1, 0)), and (P1, P2 = (1, 1)) for Design 1 of the MIMO antenna structure. Similarly, the co- and cross-polarization of the structure is shown for these port excitation conditions in Figure 7b,d,f. For all-port excitation, we’ve additionally shown the radiation pattern in 3-D distribution, as shown in Figure 7g. We have also simulated antenna structure 2 for electric field intensity and radiation patterns. Figure 8 shows these results for the multi-port excitation conditions such as (P1,P2,P3 = (1,0,0)), (P1,P2,P3 = (1,1,0)), and (P1,P2,P3 = (1,1,1)) in the form of radiation beams.

Different electric field distributions for two-port and three-port excitation are shown in Figure 9, Figure 10 and Figure 11 as co- and cross-polarization radiation patterns. It is observed that the changes in the port excitation in horizonal/vertical lines are largely affected by radiation beam generations. The radiation pattern can also be controlled by exciting the different ports at the same time. In many of the cases, the overall radiation pattern is observed as multibeam radiation. Figure 9g,h, Figure 10g,h and Figure 11g,h show the overall radiation pattern in two- and three-dimensional space, while all ports are excited for their respective antenna structures. It is also observed that the major changes in the radiation pattern occur when the top antenna structure changes from a circular patch to a split ring resonator. In the case of the scattered rectangular ground plane observed in Designs 4 and 5, the radiation pattern is distributed over all angles with less specific directional peaks under all port excitation conditions. In the case of Design 3, the radiation pattern is multibeam, which is caused by the continuous ground plane over the antenna element structures. 

## 3. MIMO Antenna Parameters

ECC is calculated using the electric field’s magnitude in Equation (1). The ECC solid angle components are indicated by ith and jth. It is time-consuming and labor-intensive to evaluate ECC using far-field radiation characteristics. Equation (2) demonstrates an alternate way of calculating ECC with the help of S-parameters, as described in [32].

(1) $$\rho_{ij}={\left|\frac{\iint \left(E_{\theta_{i}}\cdot E_{\theta_{j}}^{*}+E_{\varphi }:E_{\varphi_{j}}^{*}\right)d\Omega }{\iint \left(E_{\theta_{i}}\cdot E_{\theta_{i}}^{*}+E_{\varphi_{i}}\cdot E_{\varphi_{i}}^{*}\right)d\Omega \iint \left(E_{\theta_{j}}\cdot E_{\theta_{j}}^{*}+E_{\varphi_{j}}\cdot E_{\varphi_{i}}^{*}\right)d\Omega }\right|}^{2}$$

(2) $$\rho_{ij}=\frac{{\left|S_{11}^{*}S_{12}+S_{21}^{*}S_{22}\right|}^{2}}{\left(1-\left({\left|S_{22}\right|}^{2}+{\left|S_{12}\right|}^{2}\right)\right)\left(1-\left({\left|S_{11}\right|}^{2}+{\left|S_{21}\right|}^{2}\right)\right)}$$

**Figure 12:**
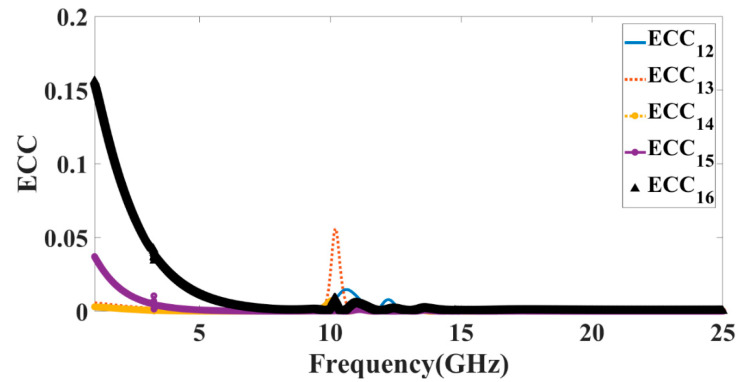
Calculated values of the ECC for antenna Design 5.

The results of the ECC calculations for antenna Design 5 are shown in Figure 12. Figure 12 shows that the antenna has an ECC of less than 0.001 for its principal frequency range of operation. Incorporating these ECC thresholds has increased the system’s robustness. When the ECC value of an antenna is low, there are fewer links between its individual parts. Due to the low numbers, it is considered that the antenna’s MIMO performance is excellent. Mixing antenna elements with different fading characteristics helps reduce the fading’s impact.

(3) $$DG=10\sqrt{1-|ECC{|}^{2}}$$

(4) $${MEG}_{i}=0.5\eta_{i,rad}=0.5\left[1-\sum_{j=1}^{M}{\left|S_{ij}\right|}^{2}\right]$$

**Figure 13:**
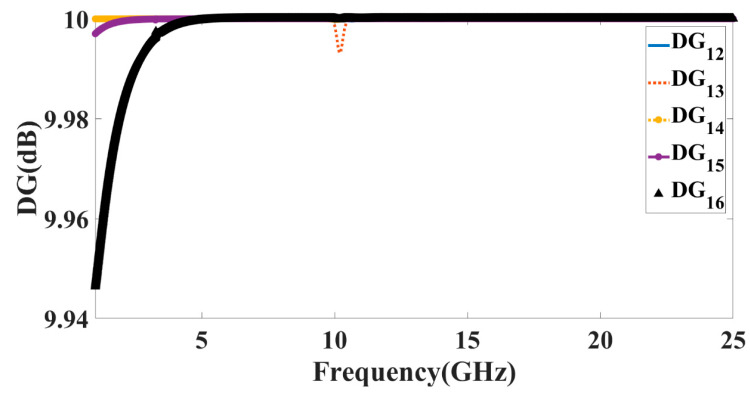
Calculated values of the DG for antenna Design 5.

For a given channel, the diversity gain [30] may be calculated by comparing the SNR of the diversity antenna system to that of a comparable single diversity antenna system. Using Equation (3), we can calculate the maximum theorized 10 dB diversity gain for a given ECC and DG. These two factors should be considered together. As diversity gain increases, multi-user MIMO antenna patches become more and more separated from one another. That being said, diversity gain’s appropriate value is at least 9 dB, as shown in Figure 13. DG values are projected to surpass 10 dB in all operating bands of both antennas. As such, it ensures that the proposed MIMO design has acceptable diversity performance. The graph of the different DG values seems to overlap with each other because of the similar response for different port excitations that has been observed in numerical calculations.

**Figure 14:**
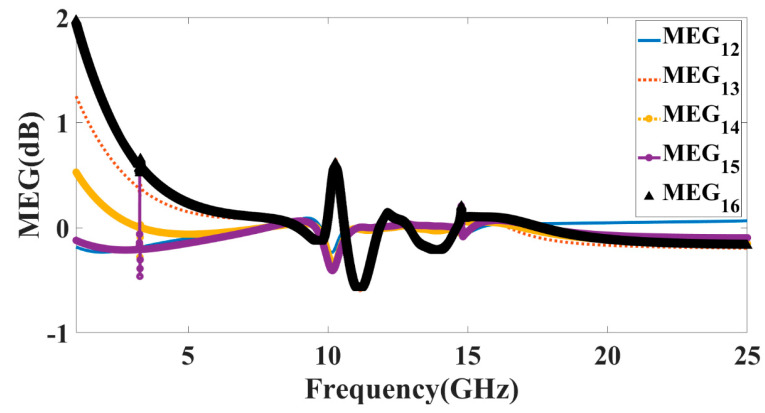
Calculated values of the MEG for antenna Design 5.

Two isotropic antennas combined average power in the absence of noise is less than or equal to the noise-averaged power delivered to a diversity antenna, as measured by the mean error gain (MEG) parameter (or interference). It demonstrates how environmental factors influence the enhanced performance of a MIMO antenna. The MEG may be validated by using Equation (4), which is based on the equation proposed in [33]. In this formula, M represents the total number of ports in the MIMO configuration, and, $\eta_{i,rad}$ is the performance in terms of radiation efficiency of the proposed MIMO configurations. Setting the mean effective gain to −3dB will maximize diversity performance on all of the device’s interfaces. In addition, there should be no more than a 0 dB divergence between the two ports. The MEG −3dB values for all three modes of operation are shown in Figure 14.

In cases where more than one port is being utilized, the total active reflection coefficient (TARC) provides the most precise measurement of radiation performance and frequency response. This is because TARC is the most trustworthy metric. The mathematical expression for determining this value is as follows: (total power incident on the item) (the total reflected power’s square root) (total power incident on the object). Using a metric called the total active reflection coefficient (TARC), researchers may determine how effective a MIMO system is at re-directing light. This methodology was developed in the 1990s. This tactic takes into consideration both the reciprocal coupling that exists on the network as well as the random pairings of signals that exist on the network. In this example, we see how the properties of both incident and reflected waves may be described using Equation (5). It suggests that one way to find out the answer to this question is by solving Equation (6) using S-parameters [34]. 

**Figure 15:**
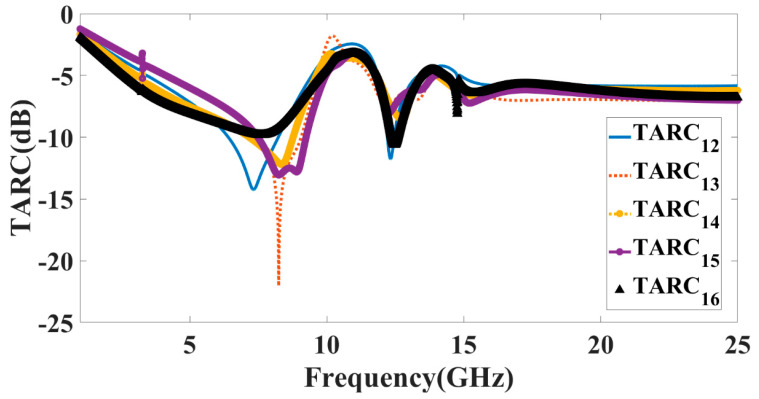
Calculated values of the TARC for antenna Design 5.

In Figure 15, we can observe the variation in TARC values produced by the two variants of the suggested MIMO antenna. The performance of the THz band MIMO data collection has been studied, and it has been determined that it satisfies the requirements of the planned usage.

(5) $$\Gamma_{a}^{t}=\frac{\sqrt{\Sigma_{j}^{M}{\left|b_{j}\right|}^{2}}}{\sqrt{\Sigma_{j}^{M}{\left|a_{j}\right|}^{2}}}$$

(6) $$\Gamma_{a}^{t}=\sqrt{\frac{{\left|S_{11}+S_{12}e^{j\theta }\right|}^{2}+{\left|S_{21}+S_{22}e^{j\theta }\right|}^{2}}{2}}$$

**Figure 16:**
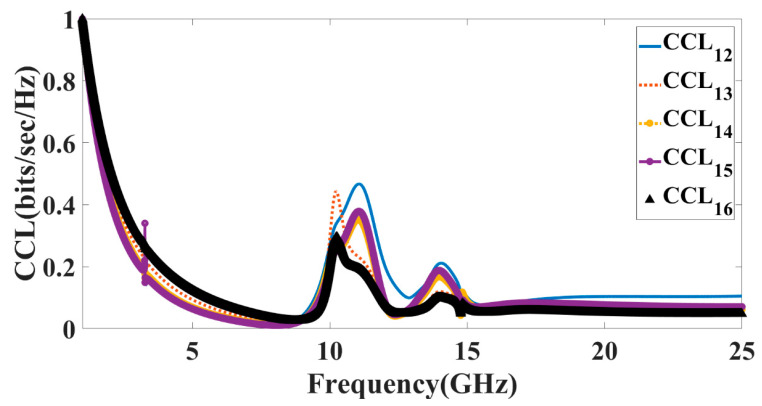
Calculated values of the CCL for antenna Design 5.

While assessing the GHz antenna’s MIMO performance, it’s also important to take into account the channel capacity loss (CCL). The greatest data transfer rate that may be achieved across a given channel without incurring a discernible quality loss is determined by the channel’s capacity loss. Rates below 0.5 bits/s/Hz are required to show that information may be sent without corruption using a well-planned MIMO system. The CCL parameter may be determined with the help of Equations (7)–(9), which can be found in [34]. As can be seen in Figure 16, this CCL limit has been reached for many bands in both configurations.

(7) $$C_{loss}=-d\;e\left(a^{R}\right)$$

(8) $$a^{R}=\left(\rho_{11}\|\|\rho_{12}\rho_{21}\|\|\rho_{22}\right)$$

(9) $$\rho_{ii}=1-\left({\left|S_{ii}\right|}^{2}+{\left|S_{ij}\right|}^{2}\right),and\;\rho_{ij}=-\left(s_{ii}^{*}S_{ij}+s_{ij}^{*}S_{ij}\right),where\;i,j\;=\;1\;or\;2$$

Our analysis involved a comparative table of the radiation element of the antenna between our computed results and those previously published, such as S parameter isolation, peak gain, bandwidth, and antenna area. The comparative analysis with other antennas is shown in Table 5. This comparative analysis shows the derived values of bandwidth, peak gain, antenna elements, and dimension for simulated and measured results with previously published data. We have identified that the proposed antenna offers a large bandwidth with better isolation values over the entire frequency spectrum. The proposed antenna also offers a large bandwidth conversion of nearly ~70% from 1 to 25 GHz of the range. The results presented in this manuscript can be applied to the various multiband satellite communication applications. 

## 4. Comparison of Massive MIMO Structure with Two Element MIMO Structure

We have identified a few advantages and disadvantages from our previously published results [9,10,35] and other published structures [36,37,38,39]. The advantages of a 1 × 2 MIMO (multiple-input single-output) structure versus a 2 × 3 MIMO structure (or massive MIMO structure) in wireless communication systems can be summarized as follows:

Advantages of 1 × 2 MIMO:

- Simplicity: A 1 × 2 MIMO structure involves one transmit antenna and two receive antennas. It is a relatively simple configuration compared to higher-order MIMO structures, such as 2 × 3, in terms of hardware complexity, implementation, and cost.
- Lower System Complexity: With fewer antennas involved, a 1 × 2 MIMO system typically has lower system complexity in terms of signal processing, antenna design, and RF (radio frequency) front-end requirements. This can result in easier system design and reduced complexity in terms of implementation and deployment.
- Lower Power Consumption: Due to its lower system complexity, a 1 × 2 MIMO structure may require less power consumption compared to higher-order MIMO configurations, which can be beneficial in scenarios where power efficiency is a concern, such as in battery-powered devices or energy-constrained environments.

Advantages of 2 × 3 MIMO:

- Higher Spatial Diversity: A 2 × 3 MIMO structure provides higher spatial diversity compared to a 1 × 2 MIMO structure. With two transmit antennas and three receive antennas, it can leverage multiple spatial paths for communication, leading to improved performance in terms of signal quality, reliability, and robustness against fading and interference.
- Increased Capacity: A 2 × 3 MIMO system can potentially offer higher capacity compared to a 1 × 2 MIMO system, as it can support the transmission of more data streams simultaneously. This can result in higher data rates and increased throughput, which can be advantageous in high-data-rate communication scenarios.
- Enhanced Performance: With more antennas involved, a 2 × 3 MIMO system can offer improved performance in terms of link quality, coverage area, and interference mitigation. It can provide better signal strength, extended coverage, and increased resilience against channel impairments, leading to more reliable and robust communication.
- Flexibility in Spatial Multiplexing: A 2 × 3 MIMO system can support higher-order spatial multiplexing, which refers to the simultaneous transmission of multiple data streams on the same frequency band. This allows for more flexibility in terms of data transmission and can enable higher capacity and improved spectral efficiency.

We can identify from the key points above that the advantages of a 1 × 2 MIMO versus a 2 × 3 MIMO structure depend on the specific requirements of the communication system and the trade-offs between system complexity, performance, and capacity. While a 1 × 2 MIMO structure offers simplicity and lower system complexity, a 2 × 3 MIMO structure provides higher spatial diversity, increased capacity, and enhanced performance. The choice between the two would depend on the specific application, deployment scenario, and performance requirements of the wireless communication system.

**Table 5:** Comparison of the results derived in the proposed antenna and previously published results.

Previously Published Result Reference	Antenna Radiation Elements	S Parameter Isolation (dB)	Peak Gain Value (dBi)	Bandwidth of the Antenna (GHz)	Antenna Dimension (Area)
This structure (Simulated)	6	~−19.03	28	10.68	7400 mm^2^
This structure (Measured)	6	~−19.68	13.71	6.1	7400 mm^2^
[36]	2	−21	4	8.6	680 mm^2^
[37]	4	−22	-	8.03	1750 mm^2^
[38]	4	−10	2.75	4	2025 mm^2^
[39]	2	−22	1	7.5	640 mm^2^
[40]	4	−10	3.1	0.2	5184 mm^2^
[41]	2	−20	3.5	7.5	4371 mm^2^
[42]	4	−15.4	1.41	5.8	676 mm^2^
[43]	4	−11	4	2.24	1600 mm^2^
[44]	4	−15	3.5	7.5	1600 mm^2^
[45]	2	−15	5.1	0.6	13,125 mm^2^
[12]	4	−15	5.5	1.1	16,800 mm^2^
[46]	4	−21	6.5	8.98	2460 mm^2^
[47]	4	−17	2.9	8.2	1600 mm^2^
[48]	4	−13	6.2	2	5625 mm^2^
[49]	4	−13	6.2	0.5	5624 mm^2^

## 5. Conclusions

This manuscript explores the use of a six-element split ring resonator with a circular patch-shaped multi-input multi-output (MIMO) antenna in the 1 to 25 GHz band and provides experimental verification of the computational results. Reflectance, gain, directivity, VSWR, and electric field distribution are only a few of the physical factors that are taken into account while analyzing MIMO antennas. The proposed antenna offers the multichannel MIMO antenna values of TARC (<−5 dB), ECC (0 to 0.15), CCL (0–1 bits/s/Hz), MEG (−1 to 1 dB), and DG (10 dB) for effective communication. The suggested antenna has a return loss of −19 dB and a gain of −28 dBi, making it suitable for ultra-wideband (10.83 GHz) applications. The antenna’s lowest return loss is −32.74 dB over its 6.89 GHz bandwidth operational range of 1.92 to 9.81 GHz. Both the continuous ground patch and the dispersed rectangular patch properties of the antennas are explored. The proposed antenna can be used for the multichannel satellite communication application with the C, X, Ku, and K bands. 

## Figures and Tables

**Table 1:** Antenna dimensions for the proposed structure of MIMO.

Term	m	l	P	g_p_	L_1_	L_2_	W_1_	W_2_	g_m_	r_1_	r_2_
(mm)	12	20	14	6	76	44	2	3	4	20	13

**Table 2:** Different split rings and circular patches shaped the proposed antenna with its fabricated images and its description of the design.

Sr. No	Design	Images of Fabricated Antenna	Description
1	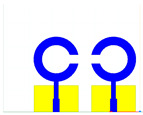	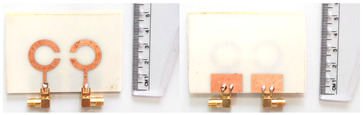	Tow element split ring patch antenna with rectangular patch
2	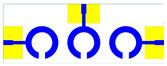	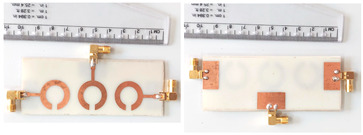	Three element split ring patch antenna
3	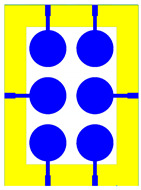	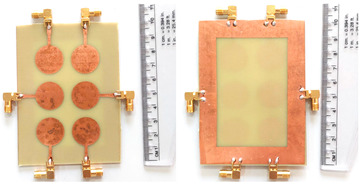	Circular patch shaped six element antennas with continuous ground
4	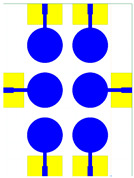	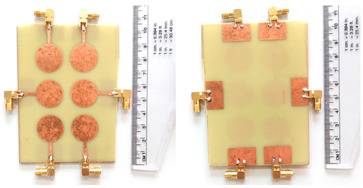	Circular patch shaped six element antennas with rectangular patch
5	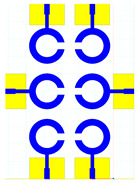	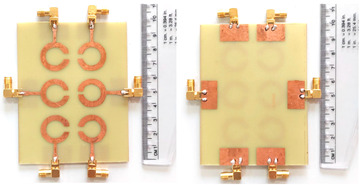	Split ring resonator shaped six element antennas with rectangular patches.

**Table 3:** Simulation based derived results and comparative analysis for the different antenna designs.

Design	Minimum Return Loss (dB)	Peak Gain (dBi)	$f_{min}$ (GHz)	$f_{max}$ (GHz)	Bandwidth (GHz)
Design 1	−34.51	9.01	2.03	9.91	7.88
	−15.84	12.17	11.78	16.95	5.17
	−16.11	12.64	19.54	25	5.46
Design 2	−25.95	21.72	4.61	9	4.39
	−15.12	5.27	11.88	14.35	2.47
	−15.48	5.184	14.95	16.33	1.38
	−22.49	16.28	18.13	22.3	4.17
Design 3	−30.26	18.92	5.45	9.24	3.79
	−28.20	16.58	14.4	16.58	2.09
	−23.70	14.56	17.45	25	7.55
Design 4	−35.80	10.23	5.42	9.57	4.15
	−17.93	10.01	11.82	14.98	3.16
	−16.16	13.13	15.94	17.33	1.39
	−28.49	17	21.96	25	3.04
Design 5	−32.74	14.74	2.92	9.81	6.89
	−15.83	4.42	11.78	13.68	1.9
	−19.68	28.23	14.32	25	10.68

**Table 4:** Measurement-based derived results and comparative analysis for the different antenna designs.

Design	Minimum Return Loss (dB)	Peak Gain (dBi)	$f_{min}$ (GHz)	$f_{max}$ (GHz)	Bandwidth (GHz)
Design 1	−24.82	13.12	4.39	10.2	5.81
	−15.62	7.35	11.82	16.77	4.35
	−19.24	12.07	20.04	25	4.96
Design 2	−21.17	11.77	4.75	8.88	4.13
	−18.22	5.81	12.4	16.37	3.97
	−26.50	16.47	18.68	24.58	5.9
Design 3	−51.90	10.59	6.15	9.72	3.57
	−20.59	19.33	14.21	15.47	1.26
	−20.17	11.59	16.87	20.80	3.93
Design 4	−22.29	22.91	4.32	9.09	4.77
	−27.25	6.08	12.33	13.12	0.79
	−15.45	13.90	14.62	18.46	3.84
Design 5	−19.03	13.71	4.20	10.30	6.1
	−14.62	5.30	12.07	13.74	1.67
	−17.23	19.53	14.37	17.69	3.32
	−27.40	15.80	19.05	25	5.95

## Data Availability

Data are available based upon a reasonable request from the corresponding author.
